# Comparative Analysis of Patient-Specific Aortic Dissections through Computational Fluid Dynamics Suggests Increased Likelihood of Degeneration in Partially Thrombosed False Lumen

**DOI:** 10.3390/bioengineering10030316

**Published:** 2023-03-01

**Authors:** Simona Moretti, Flavia Tauro, Matteo Orrico, Nicola Mangialardi, Andrea Luigi Facci

**Affiliations:** 1 DEIM Department of Economics, Engineering, Society and Business Administration, University of Tuscia, Largo dell’Università, 01100 Viterbo, Italy; 2 DIBAF Department for Innovation in Biological, Agro-Food and Forest Systems, University of Tuscia, Via San Camillo de Lellis, 01100 Viterbo, Italy; 3 Vascular and Endovascular Surgery Unit, San Camillo Forlanini Hospital, Circonvallazione Gianicolense 87, 00149 Roma, Italy

**Keywords:** CFD, Reynolds-averaged Navier–Stokes (RANS), aortic dissection, oscillatory shear index, wall shear stress, CT-based geometric models

## Abstract

Aortic dissection is a life-threatening vascular disease associated with high rates of morbidity and mortality, especially in medically underserved communities. Understanding patients’ blood flow patterns is pivotal for informing evidence-based treatment as they greatly influence the disease outcome. The present study investigates the flow patterns in the false lumen of three aorta dissections (fully perfused, partially thrombosed, and fully thrombosed) in the chronic phase, and compares them to a healthy aorta. Three-dimensional geometries of aortic true and false lumens (TLs and FLs) are reconstructed through an ad hoc developed and minimally supervised image analysis procedure. Computational fluid dynamics (CFD) is performed through a finite volume unsteady Reynolds-averaged Navier–Stokes approach assuming rigid wall aortas, Newtonian and homogeneous fluid, and incompressible flow. In addition to flow kinematics, we focus on time-averaged wall shear stress and oscillatory shear index that are recognized risk factors for aneurysmal degeneration. Our analysis shows that partially thrombosed dissection is the most prone to false lumen degeneration. In all dissections, the arteries connected to the false lumen are generally poorly perfused. Further, both true and false lumens present higher turbulence levels than the healthy aorta, and critical stagnation points. Mesh sensitivity and a thorough comparison against literature data together support the reliability of the CFD methodology. Image-based CFD simulations are efficient tools to assess the possibility of aortic dissection to lead to aneurysmal degeneration, and provide new knowledge on the hemodynamic characteristics of dissected versus healthy aortas. Similar analyses should be routinely included in patient-specific hemodynamics investigations, to plan and design tailored therapeutic strategies, and to timely assess their effectiveness.

## 1. Introduction

Among vascular diseases (VDs), aortic dissection is the detachment of the inner from the intermediate layer of the aortic wall following the creation of one or more intimal tears [1,2,3]. This generates an alternative path for blood flow called false lumen (FL) [4]. The main risk factors that prompt aortic dissection are hypertension, atherosclerosis, old age, hereditary diseases, and previous interventions such as recurrence and repair of the aneurysm [5,6]. Since the aorta is the largest artery in the body, its dissection can cause complications such as myocardial infarction, aortic rupture, and ischemia of the organs due to poor hemodynamics [5,7,8].

In developing nations and medically underserved communities, aortic dissection mortality is particularly high. For instance, in developing countries, the mortality rate is 72% within one hour after diagnosis and 92% within one week [9,10], and, among uninsured US patients, 60% of cases are detected during the autopsy and 40% suddenly die after diagnosis [11]. This evidence emphasizes the relevance of early detection, adequate follow-up in the chronic phase, and timely interventions to decrease eventual complications and fatalities.

The aorta is identified by three major divisions (ascending, descending, and abdominal), as well as the aortic arch. Several classifications of aortic dissection exist based on the extension of the disease. According to the Stanford system [12], type A dissection involves the ascending aorta while type B dissection occurs in the descending division. De Bakey et al. [13], instead, identify 3 categories: (i) in type 1, the dissection affects the whole aorta, (ii) in type 2, it involves only the ascending division, (iii) and in type 3, it is localized only in the descending aorta. Following [14], aortic dissections can also be classified according to the location of the primary entry tear. Patients with aortic dissection may exhibit either fully perfused or thrombosed (completely or partially) FLs.

Dissections involve blood flow generating stresses on the aorta walls, whereby flow patterns with higher stress levels elicit pathological processes that weaken the aorta wall and, thus, promote its aneurysmatic dilation [5,15]. High Wall Shear Stress (WSS) and its cyclic variations are key factors in dissection formation and outcome since they activate inflammatory stimuli in the inner layer endothelial cells [16,17]. In fact, high WSS values are detected close to the tear [18,19,20] and dissection breakdown in association with high pressure levels [21,22]. In addition to WSS and pressure, the Oscillatory Shear Index (OSI) is a valid predictor of WSS oscillation [23,24]. However, it does not take into account WSS intensity, which is instead synthesized through Time-Averaged WSS (TAWSS) [25].

While in-vivo flow measurements are invasive and relatively inaccurate, Computational Fluid Dynamics (CFD) allows a thorough analysis of flow patterns, kinematics, and hemodynamic forces with high spatio-temporal resolution [26]. Such analyses enlighten the physiological processes related to the disease onset and outcome, and are a valuable decision-making guide for therapists [26,27,28]. Vascular surgery may also leverage on CFD to reduce surgical trauma and blood loss [29]. While idealized geometrical models are used for general studies on aorta hemodynamics [30], refined patient-specific geometrical models are required as CFD inputs to aid in the clinical assessment of aortic hemodynamics and support dissection diagnosis [31,32].

Although the benefits of CFD have already been demonstrated in therapeutic decision making for the treatment of vascular pathologies before and after operation, no clear indication is available in the literature on the likelihood of dissection degeneration in the chronic phase. In this respect, comparative CFD investigations on the hemodynamics of dissected aortas may be beneficial to inform on the disease outcome and guide eventual surgical treatment.

Our work aims to identify critical hemodynamic features and, therefore, which dissection should undergo further treatment to avoid aneurysmal degeneration, which is the worst complication of the chronic phase of the disease [33,34]. To this end, we comprehensively reconstruct the flow distribution, kinematics, and hemodynamic forces for CT-based geometric models of three different types of dissected aorta and compare them to a healthy aorta. Namely, we investigate patient-specific models of four FLs: a chronic type B dissection, uncomplicated, treated conservatively, and currently under follow-up; a residual dissection after replacement of the ascending aorta due to type A dissection; and a chronic type B dissection which was presented as complicated in the acute phase and, thus, was treated with aortic stent grafting. We remark that, even if medical history and prognosis for type A and B dissections are different, false lumens can be considered comparable when distal from the subclavian artery. This comparative approach is foreseen to provide new knowledge on the hemodynamics of aortic dissection, thus opening novel avenues on evidence-based therapeutic treatment in the VD follow-up care. The identified critical flow patterns will be object of further research, with a larger number of patients, to gain statistical relevance.

Following a Reynolds-Averaged Navier–Stokes (RANS) approach, we identify regions of high turbulence and adopt the Q-criterion to structurally visualize turbulent flow. Our analysis allows for locating areas at high values of the WSS as well as stagnation points where blood clotting may occur. Such critical areas are indeed less oxygenated by blood motion and, therefore, the probability of rupture is higher [35].

## 2. Problem Statement

**Figure 1:**
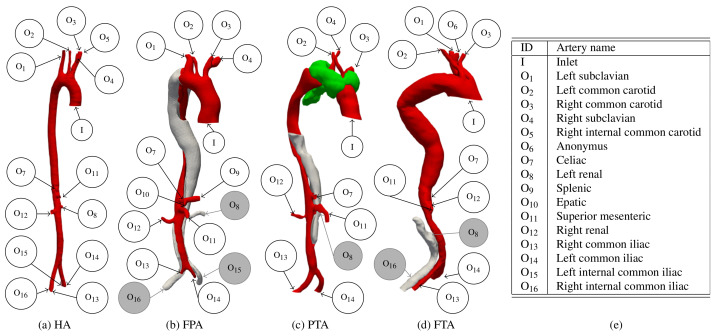
Representation of the four considered aorta models with relative arteries. TL is in red, FL is in gray and green: (**a**) HA; (**b**) FPA; (**c**) PTA where the aortic FL is in green and the abdominal FL in gray; (**d**) FTA; (**e**) names of the branches. The branches connected to the FL are highlighted in gray.

In this paper, we study the hemodynamics of the blood flow in dissected aortas using CFD. We reconstruct four patient-specific aortic models from in vivo measures: (i) a benchmark model for a healthy patient (healthy aorta, HA, Figure 1a); (ii) a model for a patient who exhibited fully perfused FL (fully perfused type B/3 aorta, FPA, Figure 1b); (iii) a model for a patient with a partially thrombosed FL (partially thrombosed type A/1 aorta, PTA, Figure 1c); and (iv) a model for a patient who developed complete FL thrombosis following thoracic endovascular aortic repair (fully thrombosed B/3 type aorta, FTA, Figure 1d). The FPA model geometry presents an FL that originates from a single tear just downstream from the aortic arch and continues down to the iliac arteries without any further connections to the true lumen (TL, in gray in Figure 1b). The FL diameter tends to be larger than the TL, especially from the aortic arch down to the abdominal branches. The PTA geometry has a FL that originates in the ascending aorta and then merges back into the abdominal TL. At the beginning of the descending aorta, the FL is thrombosed, not displayed in Figure 1c. The considerably dilated aortic arch FL (in green in Figure 1c) is fed blood from one tear up and one downstream the arch, while the abdominal TL feeds blood to the FL (in gray in Figure 1c) through five additional tears. The FL merges back into the TL three diameters above the iliac branches. Each perfused FL reach causes an abrupt reduction of the TL cross-section at the aortic arch and in proximity of the onset of the abdominal FL, respectively. Patient FTA was treated with two consistent overlapping Valiant devices (Valiant Captivia, Medtronic Vascular, Santa Rosa, California; proximal diameter: 40 mm, distal diameter: 40 mm, endoprosthesis length: 200 mm) to induce FL thrombosis from the aortic arch down to the left renal artery. Figure 1d only depicts the perfused abdominal FL in gray. The TL feeds blood to the abdominal FL through two tears just below the left renal artery. The FTA geometry presents the largest inlet section among all models, and a significant restriction at the onset of the perfused FL. In all geometries, the left renal artery originates from the abdominal FL, which was purposely left perfused in the FTA.

For this study, formal ethical approval was not required, as prior agreement was established to undertake computational modeling work using totally anonymized images without requiring further specific ethics committee agreement for individual patients. All data were analyzed anonymously and patient information was deidentified prior to analysis.

We assume the aorta rigid and large enough to consider the blood as a Newtonian and homogeneous fluid [18], while the flow can be safely considered incompressible [36]. Such a flow is modeled by the following Navier–Stokes (N-S) equation (Equation (1)) and mass conservation equation (Equation (2)):

(1) $$\rho \frac{\partial \vec{u}}{\partial t}+\rho \left(\vec{u}\;\cdot \;\nabla \right)\vec{u}=-\nabla p+\mu \nabla^{2}\vec{u}\;,$$

(2) $$\nabla \;\cdot \;\vec{u}=0\;,$$

where *t* is the time, $\vec{u}=\left(u_{x},u_{y},u_{z}\right)$ is the velocity field, *p* is pressure, $\mu =3.71\times 10^{-3}\;\mathrm{kg}/m\;s$ is the dynamic viscosity [37], and $\rho =1060\;\mathrm{kg}/m^{3}$ the blood density [38].

**Figure 2:**
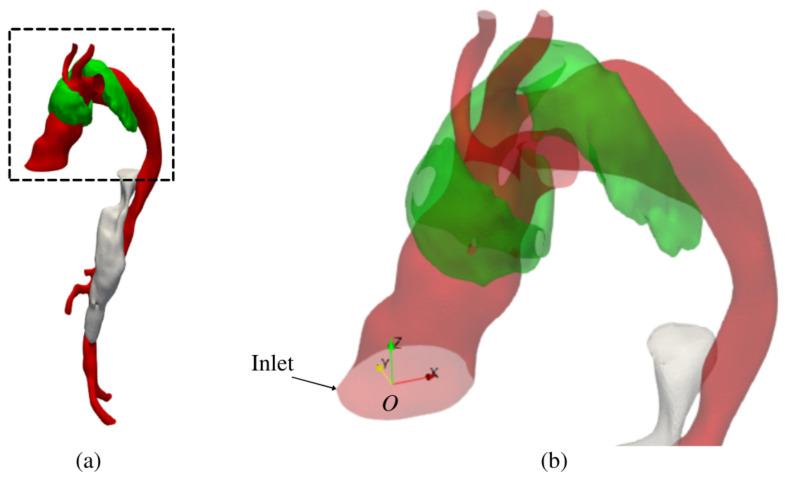
Representative 3D domain for PTA including the TL (red) and the FL (green and gray). (**a**) Complete geometry and (**b**) focus on the reference system whose origin *O* is in the centroid of the inlet section.

We select the orthogonal Cartesian reference frame O−xyz with the *z*-axis directed upward according to the direction of the flow leaving the heart through the ascending aorta, the *y*-axis according to the anterior–posterior direction, and the origin *O* located as schematized in Figure 2.

The Reynolds number, Re=uIρD/μ [37], estimated as a function of the inlet section diameter *D* and of the average inlet velocity $u_{I}$, defines the flow regime. The blood flow is pulsating [39] and 150<Re<6000[37]. With such figures, the flow varies among laminar, transitional, and turbulent regimes as a function of *t* during the cardiac cycle.

We focus on pressure and shear forces that the blood flow generates on the aorta wall as well as on coherent flow structures. Specifically, $\vec{\mathrm{WSS}}$ is determined through Equation (3) after solving Equations (1) and (2):

(3) $$\vec{\mathrm{WSS}}={\left.-\mu \frac{\partial \vec{u}}{\partial \hat{n}}\right|}_{\mathrm{wall}}\;,$$

where $\hat{n}$ is the unit vector normal to the wall [40]. In the rest of the paper, we refer to the magnitude of $\vec{\mathrm{WSS}}$ as WSS. The Wall Shear Force (WSF) is defined as:

(4) $$\vec{\mathrm{WSF}}=\int_{s}\vec{\mathrm{WSS}}\;ds\;,$$

where ds is the surface element.

We identify the vortex structures through the 2nd invariant of the velocity gradient tensor:

(5) $$Q=\frac{1}{2}\left({\left|\left|\underline{\Omega }\right|\right|}^{2}+{\left|\left|\underline{S}\right|\right|}^{2}\right)\;,$$

where $\underline{S}$ is the stress rate tensor and $\underline{\Omega }$ is the vorticity tensor. The Q-criterion defines the vortexes as connected fluid regions with a positive *Q* [41]. Finally, turbulence intensity [42]

(6) $$T=\frac{\sqrt{\frac{2}{3}}k}{u}\;,$$

describes the level of flow turbulence, being *u* the magnitude of the instantaneous local velocity, and *k*—the turbulent kinetic energy.

## 3. Methods

### 3.1. Three-Dimensional Geometry Reconstruction from CT and Models for CFD Simulation

Cephalocaudal thoracic and abdominal CT scans were executed from 2013 to 2020. Table 1 details the CT scan settings, where the field of view was consistently set to 512×512pixels, the gantry detector tilt to $0^{\circ }$, and the tube voltage (KPV) to 120kV.

**Figure 3:**
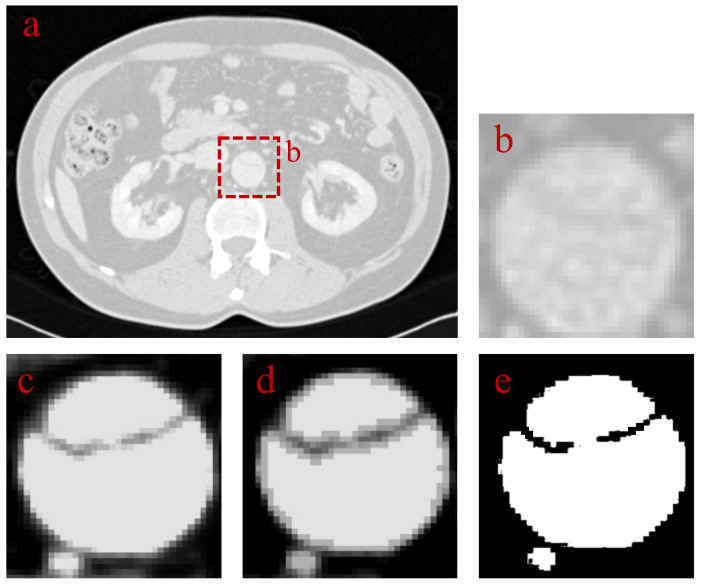
Image-based procedure for reconstructing the aortic geometric models: (**a**) example of DICOM image from the FPA CT scan sequence; (**b**) identification of the region of interest; (**c**) enhancement of image contrast; (**d**) smoothing of lumen boundaries through the ad hoc developed image filter; and (**e**) image binarization.

The patient-specific aortic geometric models are reconstructed from CT scan DICOM images [43] through an ad hoc procedure developed in the Matlab environment (Figure 3). The steps of the procedure are the following: (i) manual identification of a region of interest enclosing both true and false lumens; (ii) contrast enhancement through histogram equalization; (iii) image binarization by thresholding; and (iv) object boundary identification through the Moore Neighbor tracing algorithm modified by Jacob’s stopping criteria [44]. In case of poor visibility of the aortic dissection, image contrast is enhanced through adaptive thresholding. Namely, a threshold mask image is introduced based on a local 4-connected pixel neighborhood. Then, the mean for the local neighborhood is compared to a constant threshold: if the mean is higher than such a value, a constant value is subtracted from the central pixel of the mask. This technique affords a reduction of the background noise through smoothing of the lumen boundaries.

**Figure 4:**
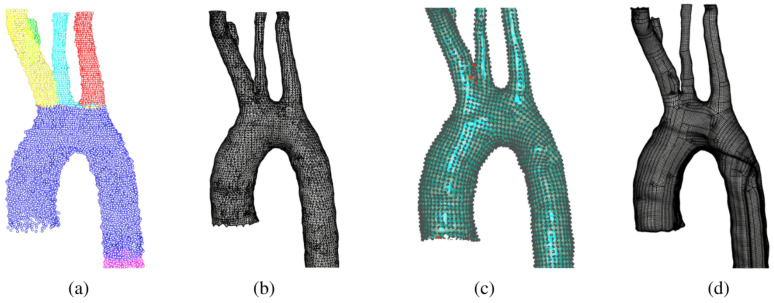
Representation of the procedure for geometry definition from point clouds to mathematical surface description. (**a**) Point cloud example; (**b**) 3D polygonal mesh resulting from points interpolation; (**c**) quadrilateral mesh obtained from Mesh2Surface with critical spots evidenced by red points; (**d**) final geometry representation.

Such a procedure returns a point cloud for each geometry (Figure 4a) that is processed through Rhinoceros v.7 to obtain mathematical models of the aorta. First, the points are interpolated through a polygonal mesh [45] (Figure 4b). Then, the Mesh2Surface Rhinoceros extension creates a structured quadrilateral surface mesh (Figure 4c) through the quadrilateral surface mesh SubD modeling technique [46]. After manual revision and cleaning of the surface features, the geometry is saved in the Initial Graphics Exchange Specification (IGES) file format [47] (Figure 4d).

### 3.2. Numerical Modelling of Blood Flow

We use the RANS [48,49,50] approach to numerically approximate Equations (1) and (2). We assume the Launder and Sharma k−ε turbulence model [51] to describe sub-grid stresses. In fact, this model is suitable to solve the low Reynolds number phenomena [51,52] (e.g., where the non-dimensional wall distance in wall units is small, $y^{+}<5$[50]) relevant in the aortic blood flow, which is in the transitional regime [48]. Specifically, it does not use any wall functions to solve the boundary layer [52], allowing a reliable evaluation of the WSS [53]. Such a closure model introduce three variables (*k*; the turbulent kinetic energy dissipation rate, ϵ; and the turbulent viscosity, $\nu_{t}$) and the relative transport equations.

Differential equations are discretized though a finite volume approach with collocated variable arrangement [54] implemented in the software package OpenFOAM-v7 [55].

The Pressure-Implicit with Splitting of Operators (PISO) algorithm [56] solves the pressure–velocity coupling and the non-linearity in Equations (1) and (2). The Euler implicit scheme [57] is selected for temporal integration. The Gauss linear scheme is used for the spatial interpolation of the gradients. The convective terms of Equations (1) and (2) are interpolated with the second-order Gauss GammaV scheme [54] with weight coefficient ϕ=0.25. Similarly, the convective terms for the *k* and ϵ equations of the Launder and Sharma model are interpolated though the Linear Upwind scheme.

The Geometric-Algebraic Multi-Grid (GAMG) iterative algorithm is used to solve the linear system of equations obtained from temporal and spatial discretization of the pressure equation, while the iterative symmetric Gauss–Seidel method solves the linear systems of equations for *u*, *k*, and ϵ [42]. Tolerances are reported in Table 2.

**Figure 5:**
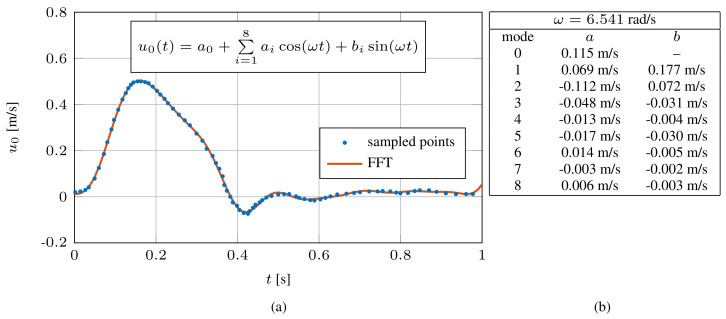
Representation of $u_{0}$ as a function of *t*: (**a**) sampled values from [38] and FFT; (**b**) coefficients of the FFT transform.

Table 3 summarizes the mathematical formulation for all the boundary conditions (BCs). In particular, a velocity inlet type BC approximates the flow that leaves the heart entering the computational domain through surface I (see the inlet identifiers in Figure 1). Specifically, $u_{0}\left(t\right)$ in Equation (8) is reported in Figure 5a. Therein, points are sampled from [38], where $u_{0}\left(t\right)$ was obtained through in vivo PC-MRI of the ascending aorta root. We use a Fast Fourier Transform (FFT)[58] to interpolate such discrete measures to the continuous periodic function also depicted in Figure 5a. The correlation coefficient is $R^{2}=0.999$ assuming 8 modes and the parameters reported in Figure 5b. We note that the peak systolic velocity is about 0.5m/s and the average inlet flow is about 6l/min [59]. The duration of a cardiac cycle is T=0.96s. The maximum Reynolds number is 4600. Note that to capture the flow kinematics during diastole, $u_{0}\left(t\right)$ allows backflow through I ($u_{0}\left(t\right)<0$ in Figure 5), see also [60].

We use inlet-outlet type BCs for all the other permeable boundaries identified by $\left[O_{1}\ldots O_{13}\right]$ in Figure 1. Such BC automatically switches between inlet and outlet modeling as a function of the flow direction [61], see Equations (9 in Table 3. Therein, $p_{O_{i}}\left(t\right)$ is determined through the 3-element Windkessel model (Equation (7), [62]) that simulates the resistance of the remaining vascular peripheral network [63,64]

(7) $$\frac{Q_{O_{i}}\left(t\right)}{C}=\frac{d(p_{O_{i}}\left(t\right)-R_{1}Q_{O_{i}}\left(t\right))}{dt}+\frac{1}{R_{2}C}\left(p_{O_{i}}\left(t\right)-R_{1}Q_{O_{i}}\left(t\right)\right)\;\;.$$

In Equation (7),$Q_{O_{i}}$ is the volume flow rate at the outlet O${}_{i}$, *C* is the network compliance, $R_{1}$ is the proximal resistance, and $R_{2}$ is the distal resistance. We estimate the constants *C*, $R_{1}$, and $R_{2}$ following the procedure in [64] and reported in Appendix A. Equation (7) is integrated in time through the Euler explicit method.

**Table 3:** Mathematical description of the assumed boundary conditions where $\hat{n}$ is the unit normal to the model surface.

Boundary	Boundary Model Equations					
I	$\vec{u}=u_{0}\left(t\right)\hat{n}$	$\frac{\partial p}{\partial \hat{n}}=0$	$k=k(t_{0})$	ϵ=ϵ(t0)∑1N		(8)
$\left[O_{1}\ldots O_{13}\right]$	$\left\{\begin{array}{c} \frac{\partial \vec{u}}{\partial \hat{n}}=0\\ \vec{u}=u_{O_{i}} \end{array}\right.$	$\left\{\begin{array}{c} p_{O_{i}}\left(t\right)\\ \frac{\partial p_{O_{i}}\left(t\right)}{\partial \hat{n}}=0 \end{array}\right.$	$\left\{\begin{array}{c} \frac{\partial k}{\partial \hat{n}}=0\\ k=k_{O_{i}} \end{array}\right.$	∂ϵ∂n^=0outflowϵ=ϵOiinflow∑1N		(9)
Wall	$\vec{u}=0$	$\frac{\partial p}{\partial \hat{n}}=0$	$k=10^{-10}$	ϵ=10−10∑1N		(10)

The aortic walls are rigid and we select the no-slip BC without wall functions, as reported by Equation (Equation (10) in Table 3.

The arteries are elongated with virtual cylinders to avoid the excessive proximity of the BCs to flow regions with significant gradients to cause numerical instability. The length of such cylinders is about 10 times the branch diameter.

We initialize the domain assuming p(t=0)=0 and $\vec{u}\left(t=0\right)=\vec{0}$. We then estimate the turbulent flow variables as:

(11a) $$\begin{array}{cc} k(t_{0}) & =\frac{1}{2}\max\left[{\left(u_{0}\left(t\right)\right)}^{2}\right]\;, \end{array}$$

(11b) $$\begin{array}{cc} \epsilon (t_{0}) & =\frac{C_{\mu }^{0.75}k^{1.5}}{L}\;, \end{array}$$

(11c) $$\begin{array}{cc} \nu_{t}\left(t_{0}\right) & =\rho C_{\mu }\frac{k^{2}}{\epsilon }\;. \end{array}$$

where L=0.038D is the reference length scale [57], and $C_{\mu }=0.09$ [51].

**Figure 6:**
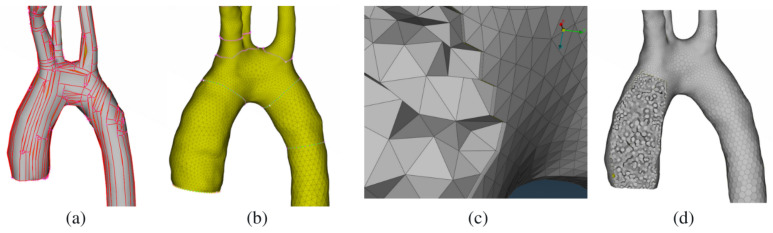
Representation of the procedure to obtain the computational mesh from the CAD model. (**a**) CAD model (i.e., mathematical surface description); (**b**) triangular surface mesh; (**c**) tetrahedral volume mesh with focus on the surface layers; (**d**) polyhedral volume mesh.

We use an unstructured meshing approach for surface and volume through the ANSA v.21.1.2 software. First, we create a triangular surface mesh using the CFD algorithm (Figure 6b), which determines the node distribution according to the curvature of the surface [65]. Specifically, we assume an interior growth rate of 1.1 and a distortion angle of 20. Then, we create 5 layers, assuming a linear growth factor of 1.2. The remaining volume is discretized using tetrahedra through the tetra-CFD algorithm with maximum growth factor of 1.1 (Figure 6c). Finally, the grid is converted into a polyhedral mesh (Figure 6d) to reduce the number of elements, improve solution convergence, and, ultimately, speed up execution [66].

Numerical simulations are performed using parallel computing through the public domain openMPI implementation of the standard message passing interface (MPI) [55]. We used the Scotch decomposition method for domain partitioning (20 sub-domains). Parallel computing was performed on 20 Intel^®^ Xeon^®^ E5-2680-v2-@-2.80 GHz processors and took between 24 h and 120 h as a function of the geometry. Equivalently, the velocity update was between 631,000 nodes per second and 850,000 nodes per second.

## 4. Convergence, Mesh Sensitivity, and Validation

### 4.1. Convergence

The assumed initial conditions significantly impact the simulation for the first cardiac cycles. We evaluate such an influence through the following mean error of the instantaneous average pressure on the surface I, $P_{I}\left(t\right)$:

(12) $$\Delta_{T}=\frac{1}{N_{s}}\sum_{l=1}^{N_{s}}\frac{P_{I}\left(t\left(l\right)\right)-P_{I}\left(t\left(l\right)-T\right)}{P_{I}\left(t\left(l\right)\right)}\;.$$

In Equation (12),$N_{s}$ is the number of samples per cycle. We perform the convergence study on the HA, whereby $\Delta_{T}<5\%$ for the 4th cycle. Thereof, we simulate the flow through all the aorta models for 4 cycles and, in the following, we present results for this type of 4th cycle.

### 4.2. Mesh Sensitivity

We also assess the impact of the mesh quality by comparing simulation results for 6 different HA domain discretizations (Table 4 and Table 5). This aims at demonstrating the independence of results from the grid.

We first analyze the mesh effects on the inlet and outlet pressures. Specifically, we compare $P_{I}$ and the average pressure P on $O_{13}$ ($P_{O_{13}}$) for meshes A and B. For instance, for $P_{I}$, the cycle average pressure difference is computed as $\Delta_{p,I}=\frac{1}{N_{s}}\sum_{l=1}^{N_{s}}\frac{|P_{I}^{A}\left(t\left(l\right)\right)-P_{I}^{B}\left(t\left(l\right)\right)|}{P_{I}^{A}\left(t\left(l\right)\right)}$. Both $\Delta_{p,I}$ and $\Delta_{p,O_{13}}$ are below or equal to 0.1%.

Finally, we assess the sensitivity of WSF on the grid as in [67] through Equation (13).

(13) $$\Delta_{\mathrm{WSF}}=\frac{1}{N_{s}}\sum_{l=1}^{N_{s}}\frac{\left|{\mathrm{WSF}}_{j}\left(t\left(l\right)\right)-{\mathrm{WSF}}_{m}\left(t\left(l\right)\right)\right|}{\max\left[\mathrm{WSF}(t(l))\right]-\min\left[\mathrm{WSF}(t(l))\right]}\;,$$

where *j* and *m* identify the different grids, and WSF is the magnitude of $\vec{\mathrm{WSF}}$. Warm colors in Table 5 indicate higher $\Delta_{\mathrm{WSF}}$. Entries in the bottom-right quadrant all exhibit cold colors, thus showing that the $y^{+}$ dominates the mesh sensitivity compared to the grid density [35]. In fact, the error is low for mesh combinations with similar $y^{+}$ and a diverse number of polyhedra (see, for example, E–F and B–C). Conversely, large $\Delta_{\mathrm{WSF}}$ are attained for mesh combinations with comparable numbers of polyhedra and different $y^{+}$ values (see, for example, C–E). Since low $y^{+}$ values yield low $\Delta_{\mathrm{WSF}}$, and a low number of polyhedra results in a smaller computational burden, we adopt mesh F as a template for all geometries.

In Table 6, we report the mesh quality evaluated through its non-orthogonality, skewness, and aspect ratio, as defined in [42]. In agreement with standard procedures in CFD for optimal solution convergence and high solution quality, the non-orthogonality is lower than 65 and the skewness is lower than 4, respectively, for all models.

### 4.3. Validation

Since to the best of our knowledge in vivo measurements are not available, we compare our results for the HA against simulation data from the literature. Specifically, we estimate $P_{I}-P_{O_{13}}≃1.6\;\mathrm{mmHg}$, which is in line with the values in [63], which are approximately 1mmHg.

Table 7 compares the percentage of inlet flow for three groups of arteries (superior, abdominal, and iliac) for our HA simulation to results from [68]. Values for the iliac flow show a remarkable agreement, while moderate differences are detected for superior and abdominal arteries. However, such differences are in line with the diverse modeling assumptions and geometries. In fact, we perform 3D simulations of real aorta models assuming rigid walls. Conversely, [68] uses a one-dimensional approach with custom BCs for 3D flow regions (e.g., junctions) and for wall elasticity, while the [69] study simplified rigid geometries.

**Figure 7:**
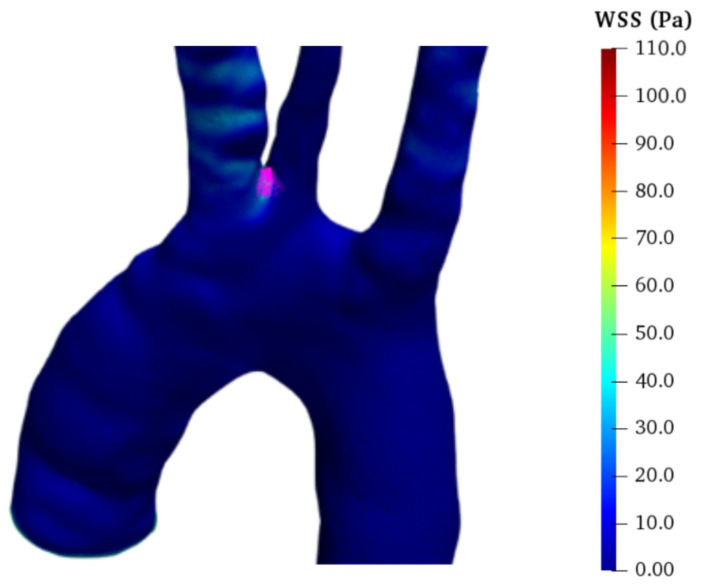
Values of WSS in the region measured by [48]. Maximum WSS values located between the subclavian and carotid roots.

Lantz et al. [48] estimate that the maximum WSS ≃45 Pa and is located between subclavian and carotid roots during systolic peak. Similarly, our simulation yields WSS ≃54 Pa in the same region as evidenced in Figure 7.

**Figure 8:**
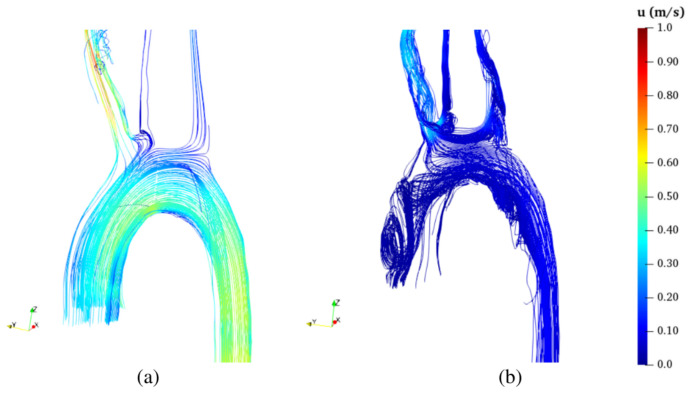
Representation of velocity streamlines in the aortic arch for the HA: (**a**) systolic peak (t=0.15s), (**b**) diastole (t=0.5s).

For a final validation, we compare a few distinctive traits of the flow patterns of HAs with references [48,60]. Figure 8a shows the streamlines in the aortic arch at the systolic peak where the uniform flow is prevalent as also highlighted in [48]. Specifically, the flow divides within the branches of the aortic arch with a pattern similar to that evidenced in [48]. At diastole, the model correctly describes the retrograde and disturbed flow patterns in the descending aorta (see Figure 8b), as reported in [48,60].

**Figure 9:**
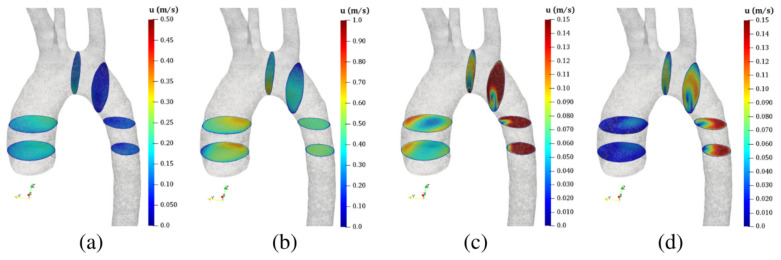
Velocity magnitude contours for the HA: (**a**) acceleration phase (t=0.1s); (**b**) systolic peak (t=0.15s); (**c**) deceleration phase (t=0.3s) and (**d**) diastole (t=0.5s).

In agreement with [48], Figure 9a,b clearly depict asymmetric flow in the ascending and uniform flow in the descending aorta, respectively. As expected, flow asymmetry in the ascending aorta is due to the superior branches flow. Likewise [48], the pattern becomes more chaotic and turbulent in the deceleration phase at time 0.3s (Figure 9c) and in the diastolic phase at time 0.5s (Figure 9d). Finally, the section between the upper arteries exhibits the same flow pattern of [48] at all time-steps (Figure 9a–d).

## 5. Results and Discussion

### 5.1. Flow Distribution

Each aortic dissection impacts the distribution of the blood volume flow through the different branches in a peculiar way (see Table 8). In the following, we quantify such impacts by computing the percentage volume flow increase/decrease for each outlet with respect to HA. In FPA, the FL drains the TL flow [38]. As a consequence, the abdominal branches (that, in this case, are connected to TL) undergo a significant flow reduction equal to 66% (O${}_{7}$), 72% (O${}_{11}$) and 88% (O${}_{12}$). Conversely, the volume flow increases by approximately 90% in the iliac branches (O${}_{15}$, O${}_{16}$) that are connected to the FL. For the PTA, the flow through the aortic arch FL reduces the flux through O${}_{3}$ by approximately 30%, while it increases the flux at O${}_{2}$ and O${}_{4}$ by 44% and 65%, respectively. Note that the left renal artery (O${}_{8}$) is connected to the abdominal FL in both PTA and FTA and its flow is reduced by 89% and 99%, respectively. Finally, for the PTA, the right renal artery flow (O${}_{12}$) is reduced by 82%, as a consequence of the abdominal FL flow that originates from two tears in the abdominal aorta region. While these findings may be patient-specific since they depend on the number and location of the intimal tears, they are well in agreement with clinical evidence. In fact, the paravisceral segment FL thrombus (as in PTA and FTA) is a frequent cause of visceral blood flow reduction in the chronic phase of aortic dissection [71].

### 5.2. Flow Kinematics

We analyse the flow kinematics for all the models at the systolic peak (t=0.15s), where the velocity is maximum, and at diastole (t=0.5s), where retrograde flow occurs in ascending aorta and the turbulence levels are higher. Further, we study in detail the streamlines patterns in the proximity of the tears, which are relevant flow regions in terms of aortic dissection outcome [72]. Finally, we dissect $u_{z}$ patterns at diastole. Enhanced-visualisation streamlines and *Q* contours are available at https://github.com/andrea-facci/Aorta (accessed on 23 January 2023) as supplementary movies.

**Figure 10:**
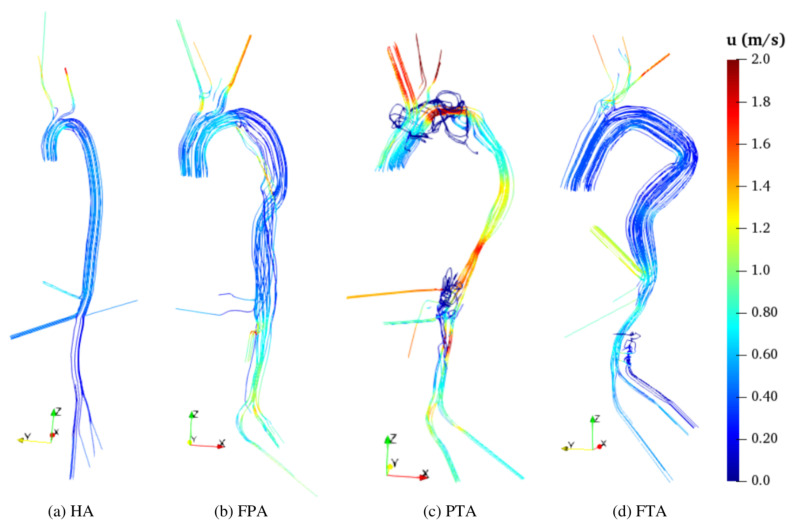
Streamlines at t=0.15s (systolic peak).

**Figure 11:**
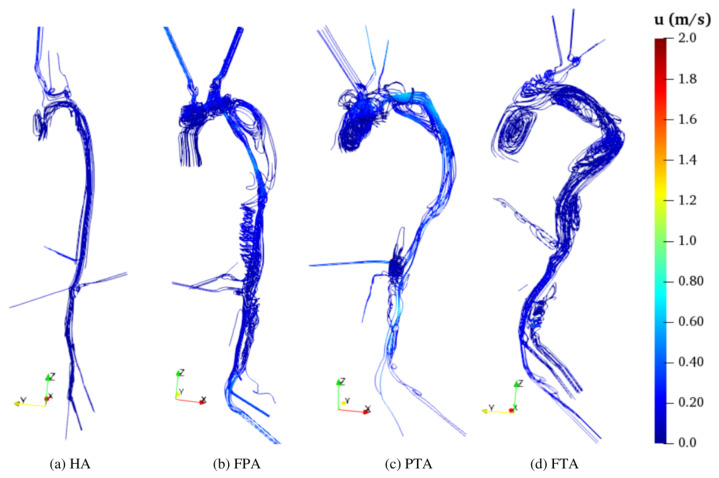
Streamlines at t=0.5s (diastole).

**Figure 12:**
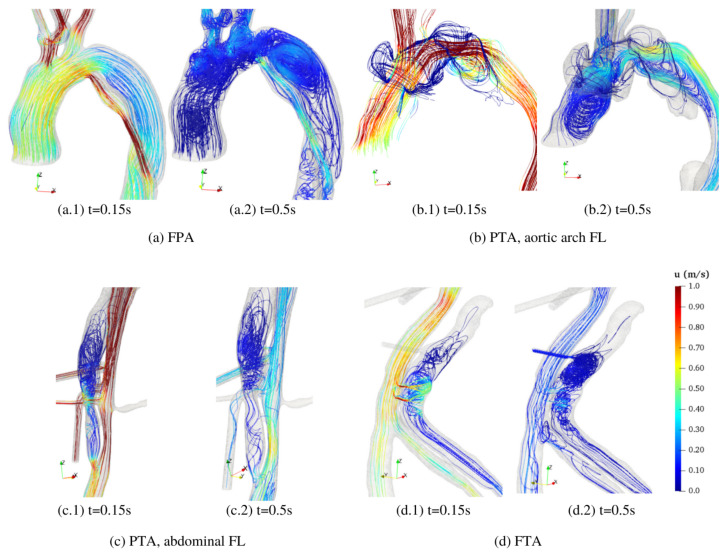
Detail of streamlines at the tears for t=0.15s (systolic peak) and t=0.5s (diastole). (**a**) Tear in the FPA; (**b**) two tears in the PTA aortic arch; (**c**) six tears in the PTA abdominal aorta; (**d**) two tears in FTA.

In all diseased aortas, the velocity pattern is less homogeneous than in HA (Figure 10 and Figure 11), and the flow accelerates along the abdominal aorta and the iliac branches (Figure 10) due to abrupt diameter variations in the TL. Such a result is more prominent in the FPA TL and in both PTA TL and FL, while the flow in the FL of the FPA (Figure 10b) and in the TL of the FTA (Figure 10d) is ordered and similar to the pattern of the HA (see also [48]). The highest velocity magnitude locates in the PTA TL that has the smallest cross-section, and three major restrictions identifiable in Figure 10c as the regions at high velocity values. One of these restrictions is located in the arch and is followed by an abrupt section enlargement that yields recirculating flow (see Figure 11c and the focused view in Figure 12b).The other two restrictions are located in the abdominal aorta. Therein, blood flow in the FL is fed from the TL exclusively through five tears located below O${}_{8}$ (Figure 12c). As a consequence, the upper part of the abdominal FL is washed by an ascending and recirculating flow as displayed by low *u* closed streamlines. Similar evidence can be found in [18]. In the FTA, the tears are also located below O${}_{8}$ (Figure 12d), which receives its flux through an ascending flow. Streamlines show a helical flow pattern of very low velocity (Figure 12c,d). This is consistent with reduced volume flows through O${}_{8}$ (see Table 8).

**Figure 13:**
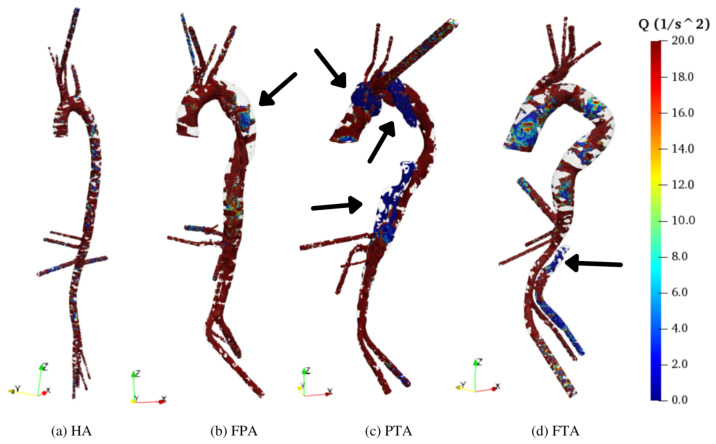
*Q* contour plots at t=0.15s (systolic peak). Gray colour represents Q<0, which are not significant [74]. Black arrows identify relevant stagnation regions.

**Figure 14:**
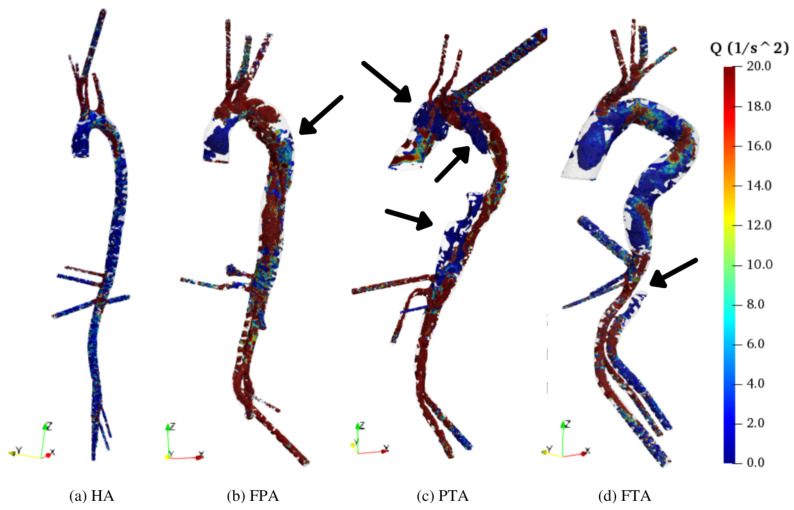
*Q* contour plots at t=0.5s (diastole). Grey colour represents Q<0, which are not significant [74]. Black arrows identify relevant stagnation regions.

In HA, the *Q* pattern is regular, with stable organized vortexes located close to the walls (Figure 13a) that represent the helical flow developing along the entire aorta (similar results are found in [60]). These structures are continuous, organized, and regular (see also [73]), and the flow pattern does not show stagnation zones (black arrows in Figure 13 and Figure 14). Moreover, such vortexes are stable in time, as arguable by comparing *Q* values for systole (Figure 13a) and for diastole (Figure 14a). However, at systole, coherent structures involve the whole domain, while, at diastole, they mainly develop along the descending and abdominal aorta. In fact, at diastole, vortex structures originate within the arch and proceed through the descending aorta [73].

In dissected aortas, coherent structures are fragmented (in particular in FPA and PTA), and stagnation regions persist in FLs during both systole and diastole, as highlighted by the arrows in Figure 13 and Figure 14. These are consistent with the rotating streamlines visible in Figure 10 and Figure 11. Specifically, FPA exhibits a single significant stagnation point that involves the aortic arch FL (Figure 13b and Figure 14b). The PTA has three dominant stagnation regions (Figure 13c and Figure 14c): two in the aortic arch FL and one in the upper portion of the abdominal FL. The FTA exhibits 25 fragmented vortex structures and a single stagnation point located in proximity of O${}_{8}$ (Figure 13d and Figure 14d).

**Figure 15:**
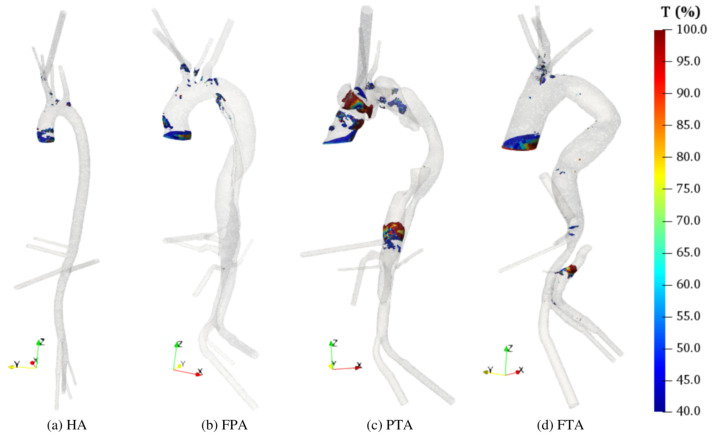
*T* contour plots at t=0.15s (systolic peak). The figure reports T>40% to improve visualization.

**Figure 16:**
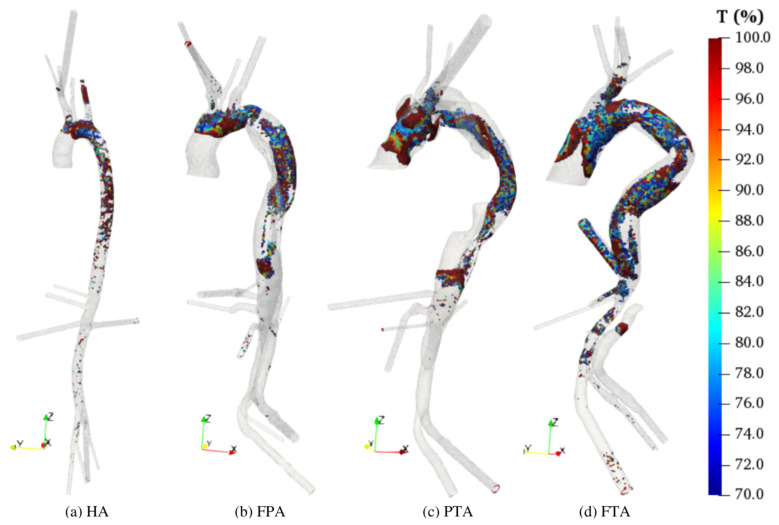
*T* contour plots at t=0.5s (diastole). For clarity, the figure reports only T>70%.

The intensity of *T* is generally higher at diastole than at systole in all geometries Figure 15. In the HA, T>70% mainly in the aortic arch (Figure 16a), consistent with the streamlines pattern in Figure 11 and the *Q* contours in Figure 14. At the systolic peak, maximum *T* is approximately equal to 40% in the ascending aorta. Small regions at higher values are located at the roots of the upper arteries. In dissected aortas, the region at T>70% is more extended than in HA, both at systole (Figure 15) and diastole (Figure 16). In the FPA, the region at higher *T* is located downstream the tear in FL at systolic peak (Figure 15b), and upstream the tear in the aortic arch at diastole (Figure 16b). In the PTA, the high *T* region is more distinctly observed at systolic peak (Figure 15c) than in the other models. Figure 15c also shows high *T* regions (≥80%) in proximity of the tears in the aortic arch and abdominal FLs. Such regions correspond to the abrupt TL restrictions. Unlike other geometries, the FTA displays high *T* also below the abdominal branches (O${}_{11}$) (Figure 15d and Figure 16d).

Our results demonstrate that the tears are major sources of highly turbulent flow, as is also clearly visible in Figure 12. For instance, Figure 12(c.2,d.2) show that the volume flow rate through O${}_{8}$ drastically drops due to the stagnating flow region. Stagnation is generated therein by the specific geometry of the FL (the top thrombosed FL is three to five FL diameters above O${}_{8}$) as well as by the relative position of O${}_{8}$ and the tear (O${}_{8}$ is approximately two FL diameters above the tear). In FPA and FTA, the flow through the tears is directed from the TL to the FL. Conversely, in the PTA, the FL bidirectionally communicates with the TL. Since the descending FL is thrombosed, the aortic arch FL feeds the TL upstream the thrombus (Figure 12b). As in the other geometries, the abdominal FL is instead fed by the TL.

**Figure 17:**
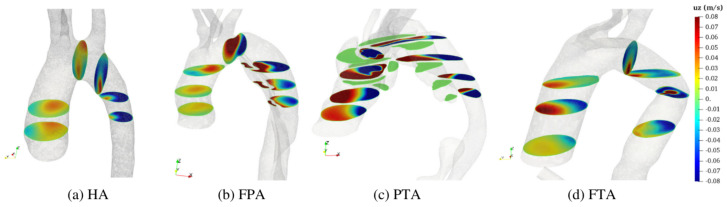
$u_{z}$ contour plots at t=0.5s (diastole). Negative values indicate backflow in the ascending aorta.

To further explore flow kinematics, we report results for $u_{z}$ at diastole in Figure 17. Such a flow pattern is generally more complicated than at systole due to the retrograde flow in ascending aorta (Figure 11). Retrograde flow is well evidenced by the $u_{z}$ contour plot in Figure 17, whereby negative values in the ascending aorta represent backflow. In the HA (Figure 17a) and FPA (Figure 17b), the pattern is similar. As expected, in the FPA aortic arch, $u_{z}$ is higher than in other models due to the TL restriction in proximity of the tear. In the PTA, $u_{z}$ is also high in the ascending aorta (Figure 17c), while it is null for a large part of the FL due to geometric constraints. Positive $u_{z}$ values in the descending aorta are a consequence of the vortexes evidenced at diastole in Figure 11.

### 5.3. Hemodynamic Forces

Hemodynamic forces are related to pressure and WSS. The pressure is higher when the flow acceleration is maximum (i.e., at t=0.1s) for all considered cases. In the HA and the FTA, the pressure is higher at the inlet and reduces towards the outlets for 0.05s<t<0.15s (i.e., when the blood flow accelerates), while it has an opposite pattern for 0.2s<t<0.4s (when the flow decelerates). On the contrary, in the FPA and PTA, the pressure reduces through the aorta also for a large part of the flow deceleration (i.e., for 0.05s<t<0.3s) due to the larger pressure drop, see the supplementary movies. In fact, in the FPA and PTA, restrictions increase the average velocity and, thus, the friction pressure losses. Moreover, vortexes identified in Figure 11 for dissected aortas are additional sources of pressure drop. In general, these considerations highlight a different balance between fluid inertia and viscous forces among the different pathologies. Specifically, for HA and FTA, inertia dominates, whereas friction dominates for FPA and PTA.

**Figure 18:**
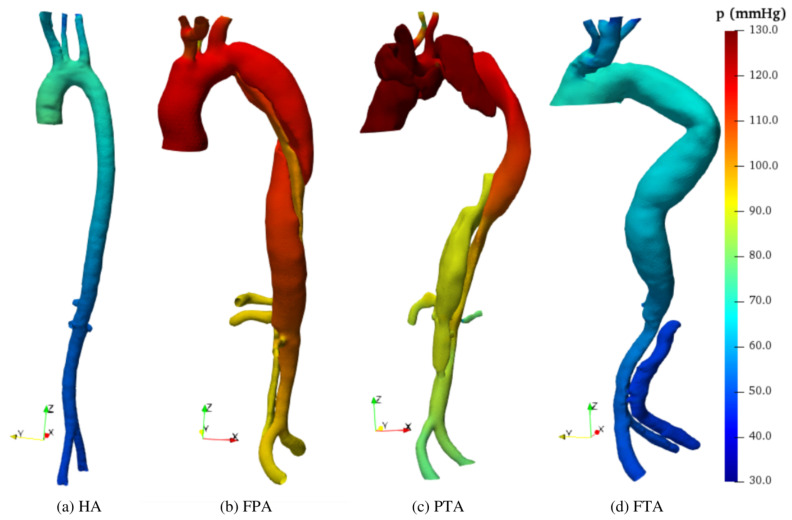
Pressure contour plots at t=0.1s (acceleration phase).

Figure 18 shows that greater *p* values locate in the ascending aorta and aortic arch (in agreement with [63]). Moreover, in FPA and PTA, *p* is significantly higher than in HA and FTA, reaching 150 mmHg for the PTA FL and 130 mmHg for the FPA. FPA and PTA have greater velocity at the iliac arteries (see Figure 10). As a consequence, in these cases, the flow back-pressure is higher, increasing the outlet pressure also for all the other branches. Specifically, the average outlet pressure is approximately 50% and 40% higher compared to the HA, for all the outlets in FPA and PTA, respectively. Conversely, in the iliac branches of the FTA, the velocity is lower compared to the HA and, in turn, the flow back-pressure is reduced by 40%. In fact, the thrombosed FL acts as a stenosis on the TL, thus reducing the distal blood flow.

We compute the average pressure drop in the cardiac cycle as $<P_{I}-P_{O_{13}}>≃1.6\;\mathrm{mmHg}$ for the HA, 4.2mmHg for the FTA, 7.3mmHg for the FPA, and 20.8mmHg for the PTA. Note that the pressure variation for the PTA is about 13 times higher compared to the HA. This probably explains the fact that PTA FL is more prone to aneurysmal degeneration and, ultimately, to aortic rupture [63]. In fact, in addition to greater pressure variations, diseased aortic walls have lower elasticity and, thus, more likely experience irreversible expansion. A preventive treatment of PTA FL may consist of surgically reaching complete FL thrombosis.

**Figure 19:**
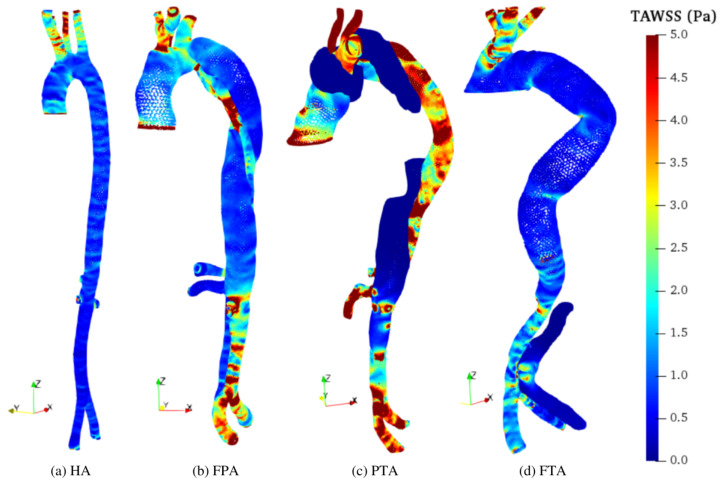
TAWSS contour plots.

Pressure drop along the aorta derives from WSS and turbulent dissipation in vortex regions. In particular, according to [23], we evaluate the time-averaged WSS (TAWSS):

(14) $$\mathrm{TAWSS}=\frac{1}{T}\int_{0}^{T}\mathrm{WSS}\;dt\;,$$

as an indicator of the total stress on the aortic wall for a complete cardiac cycle. In all models, TAWSS is large at the carotids and subclavian roots (Figure 19) where the flow section narrows, the velocity increases (Figure 10 and Figure 11), and the branch root acts as a flow obstacle. Except for such regions, in the HA, TAWSS is generally less than 2 Pa. Among dissected models, FTA has the lowest TAWSS pattern; however, moderate TAWSS values (i.e., ≃2 Pa) are detected in the abdominal TL, and greater values (>2 Pa) are found close to the tears of the abdominal FL due to the high velocities of the jet-like flow (see Figure 12 and Figure 19d). Notably, the literature identifies such areas of communication between TL and FL as potentially problematic spots [72].

In the PTA, TAWSS>2 Pa for the majority of the TL (Figure 19c), including the iliac arteries. Similar to the FTA, the jets through the communication tears between TL and FL generate high-TAWSS regions in the abdominal FL. However, the descending and aortic arch FLs exhibit the lowest TAWSS values due to the low velocity field and flow stagnation (see Section 5.2). High TAWSS values can also be observed in the FPA. Namely, TAWSS in the descending TL of the FPA is large due to the reduced flow area and consequently large velocity (Figure 19b). Similar values are detected close to the main tear in the TL aortic arch. Conversely, the FPA FL is characterized by relatively low TAWSS. However, its value increases along the abdominal FL and iliac arteries. In fact, as stated in Section 5.1, in O${}_{15}$ and O${}_{16}$, the volume flow is significantly larger compared with the other geometries.

Comprehension of the pathology evolution can further benefit from evaluation of WSS dynamics [23,35,48]. Specifically, despite low TAWSS, vortex flow regions could be prone to aneurysm formation [23] as a consequence of stress oscillation that elicits structural fatigue. The oscillatory shear index (OSI) evaluates such unsteady WSS effects [23,25]:

(15) $$\mathrm{OSI}=\frac{1}{2}\left(1-\frac{\left|\frac{1}{T}\int_{0}^{T}\vec{\mathrm{WSS}}\;dt\right|}{\frac{1}{T}\int_{0}^{T}\mathrm{WSS}\;dt}\right)\;.$$

By definition, 0≤OSI<0.5, and extended regions where OSI is close to 0.5 identify possible aneurysm formation [35].

**Figure 20:**
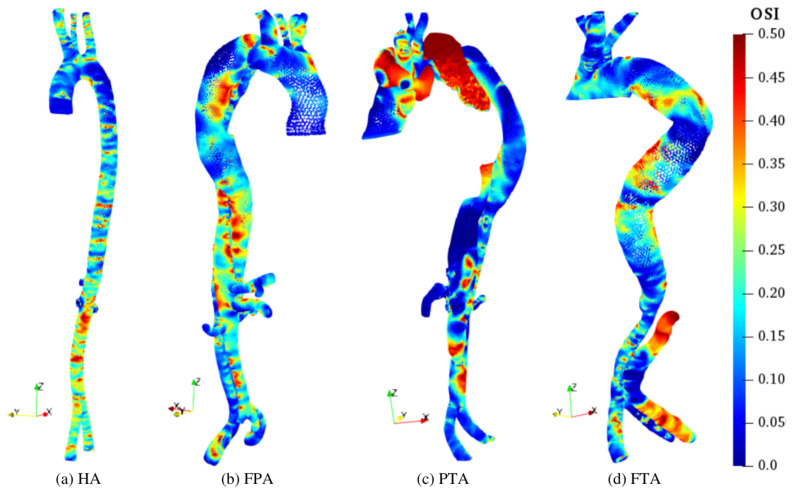
OSI contour plots.

In HA, high-OSI regions have limited extension and are generally disconnected along the abdominal aorta. Among all diseased models, the PTA exhibits the largest regions with high OSI (Figure 20c). Specifically, OSI≃0.5 for the whole aortic arch and within the upper portion of the abdominal FL, in the ascending aorta and aortic arch TL as well as where the FL merges into the abdominal TL. This is due to the presence of the stable vortex structures and stagnating flow already discussed in Secion Section 5.2. FPA shows the second most extended surface with OSI≃0.5, as reported in Figure 20b. In particular, critical regions comprise the descending and aortic arch FL and the whole TL. This can be attributed to the stable helical (vortex) flow and the irregular FL surface. Finally, in the FTA, critical regions are consistent with the locations of the vortex structures identified in Section 5.2. Specifically, the high-OSI region in the upper portion of the abdominal FL corresponds to the stagnating flow. Furthermore, regions exhibiting periodic vortexes during diastole also have high OSI values. This is in agreement with findings in [75].

## 6. Conclusions

In this paper, we provide new numerical evidence on the likelihood of degeneration in three types of aortic dissections (FPA type B/3, PTA type A/1, and FTA type B/3). We analyzed patient-specific hemodynamics and compared them to a healthy aorta (HA) by combining CT-scan images and CFD. The results show altered flow kinematics for all the dissected aortas. In particular, the branches that originate from FLs or close to tears were poorly perfused. Compared to HA, the velocity of dissected aortas was higher in the abdominal aortic reach (both for TLs and FLs) and iliac arteries. At diastole, the streamlines showed complex flow patterns characterized by high *T* and vortexes not only in the ascending but, also in the descending and abdominal aorta. According to the Q-criterion, several stagnation regions persist in all FLs and thus cause poor oxygenation that reduces the aorta wall resilience and increases the wall rupture risk [33,73].

FPA FL and PTA TL exhibit the highest pressure and TAWSS values: these are prominent risk factors for aortic degeneration and, ultimately, wall rupture [63]. In these geometries, high volume flow rate at the iliac outlets causes pressure to increase by approximately 50% in the whole domain compared to HA. Moreover, in PTA, considerable pressure losses of approximately 20mmHg during the cardiac cycle are induced by TL cross-section restrictions at the aortic arch.

Unsteady fluid forces effects further demonstrate that FPA and PTA dissections are more prone to adverse aortic evolution compared to HA. In fact, while high OSI values generally characterize all diseased geometries, the PTA and FPA exhibit the most extended coherent regions. In particular, in the PTA, the entire aortic arch FL has an OSI value close to 0.5. Since such an area also has low TAWSS values, it is more likely to experience aneurysm formation during diastole [23] and eventual subsequent rupture [33]. Conversely, the PTA FL shows low OSI values upstream from abdominal branches, thus suggesting the risk of thrombus formation [23,25]. In this scenario, our study functions as an effective decision tool supporting prognostic and therapeutic strategies. In fact, inducing the FL thrombosis, as in FTA, may effectively reduce PTA risks. Notably, FTA hemodynamic patterns more closely approximate HA, and such geometry overall exhibits less critical pressure, TAWSS, and OSI values.

Our work provides useful guidelines for future analysis of aortic dissection with CFD. Specifically, we demonstrated that the $y^{+}$ value dominates the mesh sensitivity compared with surface grid density. Thus, $y^{+}<0.3$ should be selected to correctly evaluate the WSS. Moreover, validation highlights that the RANS modeling approach, with a low-Reynolds turbulent energy equation-based closure model without wall functions, correctly balances the computational burden and modeling accuracy, yielding coherent results within acceptable computational time.

The most relevant assumptions of this study are the rigid wall and Newtonian fluid hypotheses. Elastic walls absorb wall shear stress (WSS), thus reducing its value [63]. Further, following a rigid wall approach, the effects of lumen dilatation and collapse due to pressure fluctuations are neglected. These events increase the risk of end-organ ischemia, which is not considered in our work. Nevertheless, the rigid wall assumption is widely accepted in the literature to (i) circumvent eventual uncertainties on the patient-specific wall biomechanical characteristics [25], and (ii) overcome computational costs of the fluid-structure interaction approach [39]. This study as well as numerous additional investigations [18,25,76,77,78] support the fact that the rigid wall assumption does not significantly influence aortic hemodynamic behavior, and that WSS distribution is comparable to simulation results obtained with the fluid–structure interaction approach. Regarding the Newtonian fluid assumption, we note that red blood cells cause shear thinning phenomena, where blood viscosity decreases under shear strain and high WSS values are thus reduced [79]. In the backflow phase, Newtonian models tend to overestimate the WSS and turbulent intensity [25,80]. The risk of aneurysm formation and wall rupture may in turn be underestimated in such areas where WSS values are overestimated [81,82]. Despite such criticalities, the Newtonian approximation is typically considered acceptable for large arteries, such as the aorta [83]. Generally, it is reasonable to assume a Newtonian fluid for large vessels (1–3 cm in diameter), healthy arteries and arterioles, as well as healthy veins and venules (0.2 mm–1 cm in diameter) [79]. The diameters of arteries in our models fall within those ranges spanning from 0.31 cm to 1.39 cm, and the diameter of aortas is approximately 3.5 cm. Although the number of patients included in this study is too small for statistical significance, we expect our methodology to improve in the prevention, diagnosis, and management of aortic dissection by suggesting favorable/adverse flow patterns for the dissection outcome. Further work should focus on extensively verifying such patterns for a larger number of patients. Another amelioration will entail consideration of the patient-specific circulatory system. In this study, Windkessel coefficients for all dissected geometries are consistent with those of the HA.

## Figures and Tables

**Table 1:** CT scan settings for all patients.

	HA	FPA	PTA	FTA
Age (years)	26	48	65	70
Gender	M	M	M	F
Bit depth	12	12	16	16
In-plane resolution (mm)	0.9766	0.7910	0.8125	0.8086
Slice thickness (mm)	1.5	1.0	2.5	1.25
Tube current (mA)	150	510	404	350

**Table 2:** Tolerance values for iterative linear system solvers.

Variable	Tolerance	Relative Tolerance
p/ρ	$10^{-6}\;m^{2}/s^{2}$	0.01
*u*	$10^{-6}\;m/s$	0.05
*k*	$10^{-6}\;m^{2}/s^{2}$	0.05
ϵ	$10^{-6}\;m^{2}/s^{3}$	0.05
$\nu_{t}$	$10^{-6}\;m^{2}/s$	0.05

**Table 4:** Relevant mesh properties: number of polyhedra and maximum value of the spatially averaged $y^{+}$.

Mesh	n° Polyhedra	$y^{+}$
A	310,000	1.7
B	411,000	0.93
C	600,000	0.94
D	220,000	0.56
E	706,000	0.27
F	286,000	0.24

**Table 5:** Mesh sensitivity analysis. Evaluation of $\Delta_{\mathrm{WSF}}$ in percentage for all the mesh combinations. Warm colors indicate higher errors.

Mesh	A	B	C	D	E	F
A	-	24	28	12	15	16
B	24	-	10	24	23	26
C	28	10	-	28	26	29
D	12	24	28	-	14	10
E	15	23	26	14	-	11
F	16	26	29	10	11	-

**Table 6:** Relevant quality parameters for the considered meshes.

	HA	FPA	PTA	FTA
n° surface elements	79,900	107,867	125,146	107,156
n° polyhedra	272,000	449,424	511,223	480,490
Max non-orthogonality	64.24	64.98	64.65	64.78
Max skewness	2.36	3.90	3.90	3.53
Max aspect ratio	49.1	49.9	49.9	48.5
$y^{+}$	0.239	0.243	0.296	0.125
First-layer thickness	2%	2%	2%	2%

**Table 7:** Volume flow distribution for superior, abdominal, and iliac arteries (%).

Arteries	Alastruey et al. [68]	Xiao et al. [69]	Park et al. [70]	HA
Superior	30%	21%	27%	38%
Abdominal	52%	58%	51%	44%
Iliac	18%	22%	22%	17%

**Table 8:** Volume flow distribution for the branches (%).

	$O_{1}$	$O_{2}$	$O_{3}$	$O_{4}$	$O_{5}$	$O_{6}$	$O_{7}$	$O_{8}$
HA	9.5	5.4	16.5	3.7	3.1	-	9.7	11.4
FPA	9.4	2.6	7.3	5.3	-	-	3.3	6.4
PTA	-	9.63	11.8	10.5	-	-	15.2	1.3
FTA	12.8	6.1	4.3	-	-	12.2	19.5	0.1
	$O_{9}$	$O_{10}$	$O_{11}$	$O_{12}$	$O_{13}$	$O_{14}$	$O_{15}$	$O_{16}$
HA	-	-	9.1	14	7.2	4.9	2.96	2.2
FPA	2.7	0.6	2.5	1.8	2.6	2.4	24.8	28.3
PTA	-	-	11.2	2.6	21.6	16.2	-	-
FTA	-	-	6.2	5.2	12.7	19	-	1.8

## Data Availability

Data are available at 10.5281/zenodo.5801938.

## Appendix A. Windkessel Model Implementation

We calculate the constants *C*, $R_{1}$, and $R_{2}$ of the Windkessel model following the procedure in [64]. Specifically, the proximal resistance $R_{1}$ is defined as

(A1) $$R_{1}=\frac{\rho }{A}\frac{13.3}{{\left(2r_{i}\right)}^{0.3}}\;,$$

where *A* is area of O${}_{i}$ expressed in [${\mathrm{mm}}^{2}$] and $r_{i}$ is the radius of O${}_{i}$ expressed in [mm]. Moreover, *C* is

(A2) $$C=\frac{4\times 10^{-3}\;s}{R_{t}}\;,$$

where the total resistance $R_{t}$ reads

(A3) $$R_{t}=\frac{\bar{P}}{\bar{Q}}\;,$$

where $\bar{P}$ is the mean pressure calculated following [68] and $\bar{Q}$ is the mean flow rate through O${}_{i}$. The values of $\bar{P}$ and $\bar{Q}$ are obtained from a preliminary CFD simulation where we assumed 0 back-pressure at the outlets. Finally, $R_{2}=R_{t}-R_{1}$.

Table A1, Table A2, Table A3, Table A4 summarize the values used in Equation (7).

**Table A1:** Three-element Windkessel model parameters for HA.

Output	$\bar{Q}\;[m^{3}/s$]		$\bar{P}\;[\mathrm{mmHg}$]		$R_{1}\;\left[\mathrm{kg}/m^{4}s\right]$		$R_{2}\;\left[\mathrm{kg}/m^{4}s\right]$		$C\;[m^{4}s^{2}/\mathrm{kg}]$	
$O_{1}$	$2.25\times 10^{-5}$		105.4		$2.36\times 10^{7}$		$6.02\times 10^{8}$		$2.86\times 10^{-9}$	
$O_{2}$	$1.20\times 10^{-5}$		98.9		$4.28\times 10^{7}$		$1.05\times 10^{9}$		$1.63\times 10^{-9}$	
$O_{3}$	$3.07\times 10^{-5}$		98.9		$9.21\times 10^{6}$		$3.47\times 10^{8}$		$5.03\times 10^{-9}$	
$O_{4}$	$8.37\times 10^{-6}$		99.9		$4.76\times 10^{7}$		$1.54\times 10^{9}$		$1.12\times 10^{-9}$	
$O_{5}$	$6.43\times 10^{-6}$		92.2		$6.48\times 10^{7}$		$1.85\times 10^{9}$		$9.37\times 10^{-10}$	
$O_{7}$	$8.62\times 10^{-6}$		97.8		$3.41\times 10^{7}$		$1.48\times 10^{9}$		$1.18\times 10^{-9}$	
$O_{8}$	$2.43\times 10^{-5}$		95.8		$4.08\times 10^{7}$		$4.85\times 10^{8}$		$3.40\times 10^{-9}$	
$O_{11}$	$1.97\times 10^{-5}$		97.5		$1.66\times 10^{7}$		$6.43\times 10^{8}$		$2.72\times 10^{-9}$	
$O_{12}$	$2.99\times 10^{-5}$		95.4		$9.39\times 10^{6}$		$4.16\times 10^{8}$		$4.21\times 10^{-9}$	
$O_{13}$	$1.56\times 10^{-5}$		96.9		$1.47\times 10^{7}$		$8.13\times 10^{8}$		$2.16\times 10^{-9}$	
$O_{14}$	$1.07\times 10^{-5}$		96.9		$2.33\times 10^{7}$		$1.19\times 10^{9}$		$1.48\times 10^{-9}$	
$O_{15}$	$6.31\times 10^{-6}$		96.9		$4.08\times 10^{7}$		$1.98\times 10^{9}$		$8.84\times 10^{-10}$	
$O_{16}$	$4.75\times 10^{-6}$		96.9		$5.03\times 10^{7}$		$2.64\times 10^{9}$		$6.65\times 10^{-10}$	

**Table A2:** Three-element Windkessel model parameters for FPA model.

Output	$\bar{Q}\;[m^{3}/s$]		$\bar{P}\;[\mathrm{mmHg}$]		$R_{1}\;\left[\mathrm{kg}/m^{4}s\right]$		$R_{2}\;\left[\mathrm{kg}/m^{4}s\right]$		$C\;[m^{4}s^{2}/\mathrm{kg}]$	
$O_{1}$	$1.05\times 10^{-5}$		105.4		$2.25\times 10^{7}$		$1.32\times 10^{9}$		$1.33\times 10^{-9}$	
$O_{2}$	$2.74\times 10^{-6}$		98.9		$8.87\times 10^{7}$		$4.73\times 10^{9}$		$3.72\times 10^{-10}$	
$O_{3}$	$7.78\times 10^{-6}$		99.7		$2.33\times 10^{7}$		$1.68\times 10^{9}$		$1.05\times 10^{-9}$	
$O_{4}$	$5.91\times 10^{-6}$		99.9		$5.84\times 10^{7}$		$2.19\times 10^{9}$		$7.95\times 10^{-10}$	
$O_{8}$	$6.57\times 10^{-6}$		95.8		$3.84\times 10^{7}$		$1.91\times 10^{9}$		$9.21\times 10^{-10}$	
$O_{9}$	$2.66\times 10^{-6}$		93.4		$2.46\times 10^{7}$		$4.66\times 10^{9}$		$3.82\times 10^{-10}$	
$O_{10}$	$6.69\times 10^{-7}$		97.3		$1.26\times 10^{8}$		$1.93\times 10^{10}$		$9.24\times 10^{-11}$	
$O_{11}$	$2.66\times 10^{-6}$		97.5		$5.35\times 10^{7}$		$4.84\times 10^{9}$		$3.66\times 10^{-10}$	
$O_{12}$	$1.82\times 10^{-6}$		95.4		$9.88\times 10^{7}$		$6.90\times 10^{9}$		$2.56\times 10^{-10}$	
$O_{13}$	$2.69\times 10^{-6}$		96.9		$6.59\times 10^{7}$		$4.74\times 10^{9}$		$3.72\times 10^{-10}$	
$O_{14}$	$2.52\times 10^{-6}$		96.9		$6.89\times 10^{7}$		$5.06\times 10^{9}$		$3.49\times 10^{-10}$	
$O_{15}$	$2.62\times 10^{-5}$		96.9		$1.53\times 10^{7}$		$4.78\times 10^{8}$		$3.63\times 10^{-9}$	
$O_{16}$	$2.93\times 10^{-5}$		96.9		$1.55\times 10^{7}$		$4.25\times 10^{8}$		$4.06\times 10^{-9}$	

**Table A3:** Three-element Windkessel model parameters for PTA model.

Output	$\bar{Q}\;[m^{3}/s$]		$\bar{P}\;[\mathrm{mmHg}$]		$R_{1}\;\left[\mathrm{kg}/m^{4}s\right]$		$R_{2}\;\left[\mathrm{kg}/m^{4}s\right]$		$C\;[m^{4}s^{2}/\mathrm{kg}]$	
$O_{2}$	$1.44\times 10^{-5}$		98.9		$9.27\times 10^{7}$		$8.25\times 10^{8}$		$1.95\times 10^{-9}$	
$O_{3}$	$1.57\times 10^{-4}$		98.9		$6.91\times 10^{6}$		$7.72\times 10^{7}$		$2.13\times 10^{-8}$	
$O_{4}$	$1.39\times 10^{-5}$		99.9		$7.97\times 10^{7}$		$8.82\times 10^{8}$		$1.86\times 10^{-9}$	
$O_{7}$	$1.92\times 10^{-5}$		97.8		$2.77\times 10^{7}$		$6.51\times 10^{8}$		$2.64\times 10^{-9}$	
$O_{8}$	$1.59\times 10^{-6}$		95.8		$2.21\times 10^{8}$		$7.79\times 10^{9}$		$2.23\times 10^{-10}$	
$O_{11}$	$1.41\times 10^{-5}$		97.5		$4.58\times 10^{7}$		$8.74\times 10^{8}$		$1.95\times 10^{-9}$	
$O_{12}$	$3.14\times 10^{-6}$		95.4		$1.16\times 10^{8}$		$3.93\times 10^{9}$		$4.42\times 10^{-10}$	
$O_{13}$	$2.66\times 10^{-5}$		96.9		$1.24\times 10^{7}$		$4.73\times 10^{8}$		$3.69\times 10^{-9}$	
$O_{14}$	$1.98\times 10^{-5}$		96.9		$1.48\times 10^{7}$		$6.38\times 10^{8}$		$2.74\times 10^{-9}$	

**Table A4:** Three-element Windkessel model parameters for FTA model.

Output	$\bar{Q}\;[m^{3}/s$]		$\bar{P}\;[\mathrm{mmHg}$]		$R_{1}\;\left[\mathrm{kg}/m^{4}s\right]$		$R_{2}\;\left[\mathrm{kg}/m^{4}s\right]$		$C\;[m^{4}s^{2}/\mathrm{kg}]$	
$O_{1}$	$3.38\times 10^{-5}$		105.4		$2.20\times 10^{7}$		$3.93\times 10^{8}$		$4.31\times 10^{-9}$	
$O_{2}$	$1.64\times 10^{-5}$		98.9		$5.00\times 10^{7}$		$7.52\times 10^{8}$		$2.23\times 10^{-9}$	
$O_{3}$	$2.10\times 10^{-5}$		98.9		$3.91\times 10^{7}$		$5.90\times 10^{8}$		$2.85\times 10^{-9}$	
$O_{6}$	$7.42\times 10^{-6}$		99.9		$8.56\times 10^{7}$		$1.71\times 10^{9}$		$9.97\times 10^{-10}$	
$O_{7}$	$7.88\times 10^{-5}$		97.8		$7.66\times 10^{6}$		$1.58\times 10^{8}$		$1.08\times 10^{-8}$	
$O_{8}$	$4.10\times 10^{-7}$		95.8		$1.49\times 10^{7}$		$3.11\times 10^{10}$		$5.75\times 10^{-11}$	
$O_{11}$	$1.41\times 10^{-5}$		97.5		$5.26\times 10^{7}$		$8.70\times 10^{8}$		$1.94\times 10^{-9}$	
$O_{12}$	$1.07\times 10^{-5}$		95.4		$5.10\times 10^{7}$		$1.14\times 10^{9}$		$1.51\times 10^{-9}$	
$O_{13}$	$2.19\times 10^{-5}$		96.9		$1.86\times 10^{7}$		$5.72\times 10^{8}$		$3.03\times 10^{-9}$	
$O_{14}$	$4.41\times 10^{-5}$		96.9		$8.50\times 10^{6}$		$2.84\times 10^{8}$		$6.12\times 10^{-9}$	
$O_{16}$	$6.12\times 10^{-6}$		96.9		$1.49\times 10^{7}$		$2.09\times 10^{9}$		$8.48\times 10^{-10}$	
